# Epigenetically maintained SW13+ and SW13- subtypes have different oncogenic potential and convert with HDAC1 inhibition

**DOI:** 10.1186/s12885-016-2353-7

**Published:** 2016-05-17

**Authors:** McKale R. Davis, Juliane J. Daggett, Agnes S. Pascual, Jessica M. Lam, Kathryn J. Leyva, Kimbal E. Cooper, Elizabeth E. Hull

**Affiliations:** Department of Biomedical Sciences, Midwestern University, Glendale, AZ USA; Department of Microbiology and Immunology, Midwestern University, Glendale, AZ USA

**Keywords:** Epigenetic regulation, Histone modification, HDAC inhibitors, Adrenocortical carcinoma, SWI/SNF, BRM, Chromatin remodeling

## Abstract

**Background:**

The BRM and BRG1 tumor suppressor genes are mutually exclusive ATPase subunits of the SWI/SNF chromatin remodeling complex. The human adrenal carcinoma SW13 cell line can switch between a subtype which expresses these subunits, SW13+, and one that expresses neither subunit, SW13-. Loss of BRM expression occurs post-transcriptionally and can be restored via histone deacetylase (HDAC) inhibition. However, most previously used HDAC inhibitors are toxic and broad-spectrum, providing little insight into the mechanism of the switch between subtypes. In this work, we explore the mechanisms of HDAC inhibition in promoting subtype switching and further characterize the oncogenic potential of the two epigenetically distinct SW13 subtypes.

**Methods:**

SW13 subtype morphology, chemotaxis, growth rates, and gene expression were assessed by standard immunofluorescence, transwell, growth, and qPCR assays. Metastatic potential was measured by anchorage-independent growth and MMP activity. The efficacy of HDAC inhibitors in inducing subtype switching was determined by immunofluorescence and qPCR. Histone modifications were assessed by western blot.

**Results:**

Treatment of SW13- cells with HDAC1 inhibitors most effectively promotes re-expression of BRM and VIM, characteristic of the SW13+ phenotype. During treatment, hyperacetylation of histone residues and hypertrimethylation of H3K4 is pronounced. Furthermore, histone modification enzymes, including HDACs and KDM5C, are differentially expressed during treatment but several features of this differential expression pattern differs from that seen in the SW13- and SW13+ subtypes. As the SW13- subtype is more proliferative while the SW13+ subtype is more metastatic, treatment with HDACi increases the metastatic potential of SW13 cells while restoring expression of the BRM tumor suppressor.

**Conclusions:**

When compared to the SW13- subtype, SW13+ cells have restored BRM expression, increased metastatic capacity, and significantly different expression of a variety of chromatin remodeling factors including those involved with histone acetylation and methylation. These data are consistent with a multistep mechanism of SW13- to SW13+ conversion and subtype stabilization: histone hypermodification results in the altered expression of chromatin remodeling factors and chromatin epigenetic enzymes and the re-expression of BRM which results in restoration of SWI/SNF complex function and leads to changes in chromatin structure and gene expression that stabilize the SW13+ phenotype.

**Electronic supplementary material:**

The online version of this article (doi:10.1186/s12885-016-2353-7) contains supplementary material, which is available to authorized users.

## Background

The human adrenal cortical adenocarcinoma SW13 cell line exists in two subtypes, originally distinguished by expression of the intermediate filament protein vimentin [1]. Subsequently, it was shown that the more abundant SW13- subtype, in addition to lacking vimentin, does not express either BRM (SMARCA2) or BRG1 (SMARCA4) [2]. As these proteins are the mutually exclusive ATPase subunits of the SWI/SNF chromatin remodeling complex [3], SW13- cells are presumed to have a non-functional SWI/SNF (or BAF) complex.

BRM and BRG1 are both well characterized tumor suppressors. While BRM silencing in primary tumors and in cancer cell lines is frequently epigenetic in nature, BRG1 is frequently mutated but may also be epigenetically silenced (reviewed in [4–7]). In addition, recent proteomic analysis has revealed that several other subunits of the SWI/SNF complex also function as tumor suppressors. SNF5 (SMARCB1 or BAF47) is mutated in 100 % of malignant rhabdoid tumors [8, 9]. Overall, components of the SWI/SNF complex are mutated in approximately 20 % of cancers [10, 11] suggesting that appropriate function of the entire complex is an important regulator of oncogenesis.

Switching between the SW13 subtypes was originally characterized as spontaneous but, when they are isolated by dilution cloning, the subtypes are stable for greater than 20 doublings [12]. Subsequently, it was shown that the switch from SW13- to SW13+ could be triggered by addition of trichostatin A (TSA), a broad-spectrum hydroxamic acid analog HDAC inhibitor, or its derivative CHAP31 [12]. However, these data provide little insight into the mechanism of the switch between SW13 subtypes. Subsequent work extended the study of HDAC inhibitors in the restoration of BRM activity but focused on one activity of BRM: the requirement of BRM to activate transcription downstream of glucocorticoid receptor signaling [13].

The expression of HDACs is dysregulated in many cancers, expression correlates with prognosis, and knockdown of individual HDACs leads to apoptosis in a variety of tumors (reviewed [14]). HDAC inhibitors FK228 (Romidepsin) and SAHA (Vorinostat) are FDA approved for the treatment of cutaneous T cell lymphoma. Although several other HDAC inhibitors are in clinical trials, none to date show success in the treatment of solid tumors (reviewed in [15, 16]). With the increased interest in HDAC inhibition in cancer treatment, we investigated the involvement of HDAC inhibitors in the SW13- to SW13+ subtype switch more fully, only selecting HDAC inhibitors with defined HDAC targets and minimal toxicity as evidenced by progress in clinical trials. We also characterized the oncogenic, metastatic, and epigenetic differences between the SW13 subtypes.

The work presented here focused on characterizing the SW13- to SW13+ subtype conversion as initiated by HDACi by assessing the relative efficacy of a panel of inhibitors. In order to put this epigenetically mediated subtype switch in context, we explored the starting and the ending point of the SW13- to SW13+ subtype switch by addressing differences in oncogenic potential and gene expression between the two subtypes which have been isolated by dilution cloning. Thus, the data presented here investigates both the mechanism of the HDACi mediated SW13- to SW13+ switch and the epigenetic maintenance of the two stable subtypes.

Specifically, data presented here suggest that inhibitors of HDAC1 most effectively promote re-expression of BRM and VIM characteristic of the SW13+ phenotype and do so in a dose-dependent manner. As the SW13- subtype is more proliferative while the SW13+ subtype is more metastatic, treatment with HDACi increases the metastatic potential of SW13 cells while restoring expression of the BRM tumor suppressor. In addition to hyperacetylation of histone residues, HDACi treatment of SW13- cells for 24 h results in hypertrimethylation of H3K4 and differential expression of several histone modification enzymes including several HDAC enzymes. However, when the expression patterns of these enzymes in treated SW13- cells is compared to that seen in SW13- and SW13+ subtypes isolated by dilution cloning, significant differences are seen. These data suggest that many of the changes induced by treatment of the SW13- subtype with HDACi for 24 h are not maintained in the stable subtypes after removal of HDACi.

This work has important implications for two levels of cancer biology. First, the epigenetically controlled differences between subtypes in the SW13 line may shed light on tumor heterogeneity and serve as a model system to study therapeutic approaches in a cell line whose oncogenic potential is controlled via epigenetic mechanisms. Second, given the focus on HDACi in a variety of clinical trials, the response of an adrenocortical carcinoma line to treatment with HDACi has wider implications. The ability of HDAC inhibitors to initiate a conversion between SW13- to SW13+ subtypes provides an opportunity to probe the epigenetically controlled conversion which was previously characterized as “spontaneous” but can be initiated in the SW13- to SW13+ direction by HDACi treatment.

## Methods

### Cell culture

**Fig. 1:**
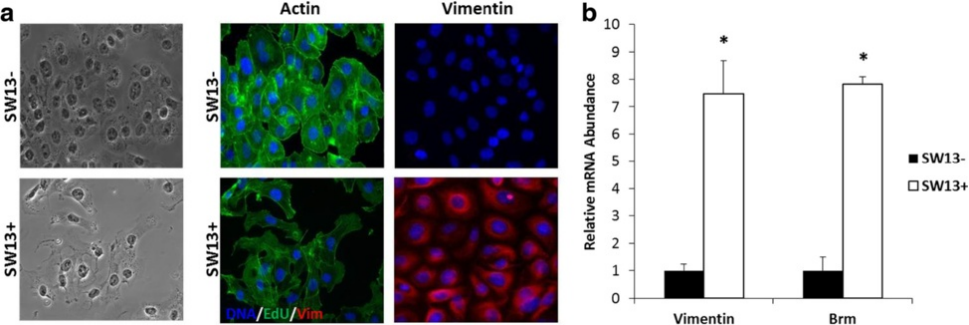
SW13- and SW13+ subtype characterization: differences in morphology, actin organization, vimentin expression, and *VIM* and *BRM* levels. **a** Subtypes have distinct morphology, actin organization, and levels of vimentin expression. *Left-hand* panel: light microscopy photographs; *Middle* panel: visualization of actin filaments with fluorescent phalloidin; *Right-hand* panel: expression of vimentin by immunofluorescence. Images were taken using a 40× oil-immersion objective lens. **b** qPCR reveals *VIM* and *BRM* mRNA expression is ~ 8 -fold higher in the SW13+ cells compared to the SW13- cells. Data are presented as mean ± SEM. *Denotes statistical difference between subtypes, *p* <0.05

SW13 cells were obtained from American Type Culture Collection (ATCC; CCL-105) and maintained in high glucose DMEM supplemented with 10 % fetal bovine serum and 10 U/ml penicillin/streptomycin at 37 °C in a humidity controlled incubator. Pure cultures of SW13+ and SW13- cells were isolated by dilution cloning and screened for vimentin expression (See Fig. 1a). All experiments were performed with early passage clones and were conducted between passages 5 and 20.

### Assessment of cell growth and proliferation

Cells were seeded at 1 × 10^4^ cells per/ml into six well plates and counted daily using a hemocytometer and trypan exclusion for 7 days. To assess differences in subtype proliferation SW13+ and SW13- cells were seeded into 8-well chamber slides at 0.5 × 10^4^ cells/ml. After 24 h, 10 μM Click-iT EdU reagent (ThermoFisher) was added to each chamber and cells were allowed to grow for another 24 h. Cells were then fixed and permeabilized and nuclear staining and EdU detection were performed according to the manufacturer’s recommendations. To assess the effects of HDACi treatment on cell proliferation, SW13- cells were treated with either 0.51 μM MS-275 or 2 nM FK228 for 24 h before labeling with EdU. Cells were imaged using a Zeiss Axiovert Apotome (Zeiss) with uniform exposure at each wavelength in each experiment. NIH ImageJ software was used to determine percent proliferation by dividing the number of cells which stained positive for EdU by the number of total cells per each group.

### Soft agar assays

Cells were plated at 5 × 10^3^ cells/well in a 0.4 % agarose/1× media solution on top of a 0.5 % agarose/1× media base layer in 6-well plates and maintained as above for 14 days replacing the media twice per week. Cells were fixed and stained overnight with a 2 % paraformaldehyde/0.005 % crystal violet solution and de-stained with water. Colonies were photographed with an AlphaImager, and colony number and size were determined using NIH ImageJ software.

### Transwell assays

SW13+ and SW13- cells were serum starved for 24 h then plated at 1 × 10^5^ cells/well in serum free media into Nunc (Thermo Scientific) cell culture inserts with 8 μm pore size polycarbonate membranes. Medium supplemented with 10 % fetal bovine serum was placed in the outer reservoir for use as a chemoattractant. Cells were incubated under standard conditions for 24 or 48 h after which the medium was removed, and the cells were fixed and stained with 2 % paraformaldehyde/0.005 % crystal violet before visualization using an Olympus microscope.

### In situ zymography

Cells were plated at 1 × 10^4^ cells/well in 8-chamber slides and MMP activity was assessed as previously described [17]. Briefly, DQ gel substrate (Life Technologies) was diluted to 40 μg/ml in MMP activity buffer (100 mM NaCl, 100 mM Tris-HCl, pH 7.5, 10 mM CaCl_2_, 20 μM ZnCl, 0.05 % NP40) with sodium azide to a concentration of 0.2 mM. Next, 200 μl diluted DQ gel substrate was added to each well and incubated under standard cell culture conditions overnight. Cells were washed 3 times with 1× phosphate buffered saline before fixation and DAPI staining. Total fluorescence was normalized to total cell number in each well. All experiments were performed at least in triplicate.

### Quantification of subtype switching

SW13- cells were treated with 3 μg/ml Scriptaid (Calbiochem), 3 μg/ml Nullscript (Biomol International), 2 μg/ml Depudecin (Enzo Life Sciences), 4 μg/ml BML 210 (Enzo Life Sciences), 1 μg/ml MS-275 (Selleckchem), 3 μg/ml MC1293 (Enzo Life Sciences), 0.6 μg/ml MGCD0103 (Selleckchem), or 0.7 μg/ml FK228 (Selleckchem) for 0, 24, 48, and 72 h before fixation and screening for vimentin expression to indicate subtype switching. NIH ImageJ software was used to determine the ratio of vimentin intensity normalized to cell number by dividing by the DAPI intensity using images photographed with equal exposures.

### Immunofluorescence

Immunofluorescence was performed using standard techniques. Specifically, for SW13-/SW13+ morphology and subtype analysis, cells were fixed with 4 % paraformaldehyde, permeabilized with 0.2 % Triton-X, and blocked with 1 % BSA before incubation with a 1:1000 dilution of a Cy3 conjugated anti-vimentin antibody (Sigma) and 25 U/ml Alexafluor 488 phalloidin (Invitrogen). Samples were photographed using a Zeiss Axiovert Apotome with uniform exposure at each wavelength in each experiment. To quantify vimentin expression after treatment with HDAC inhibitors, photographs were taken using a 10× objective lens with a constant exposure time and the ratio of vimentin to DAPI signal of ten complete fields were averaged per experiment.

### RNA isolation & quantitative real-time PCR

Cells were plated at 1 × 10^5^ into 6-well plates, allowed to adhere and grow for 24 h before indicated treatment doses and times. Following treatment, total RNA was isolated from cells using Trizol (Life Technologies) according to the manufacturer’s instructions. Following DNAse treatment, RNA was reverse-transcribed using SuperScript II (Life Technologies). Real-time PCR was performed on an ABI StepOne Plus thermocycler (Applied Biosystems) using SYBR Green Chemistry. Primer sequences used were as follows: GAPDH – Forward: 5′- ttgttgccatcaatgaccc-3′, Reverse: 5′-cttcccgttctcagccttg-3′; BRM – Forward: 5′- gattgtagaagacatccattgtgg-3′, Reverse: 5′- gacatataaccttggctgtgttga-3′; VIM – Forward 5′- gctcgtcaccttcgtgaata-3′, Reverse 5′- tcgttgataacctgtccatctc-3′. Relative mRNA expression was determined using the 2^-ΔΔCt^ method with GAPDH as the invariant control.

### Pathway-focused PCR arrays

For the pathway-focused PCR arrays, cDNA synthesis was performed using 0.5 μg total RNA and SABiosciences RT^2^ First Strand cDNA kit according to the kit instructions. Profiler PCR Array System by SABiosciences following the manufacturer’s instructions. Quantitative real-time PCR was done using the Human Epigenetic Chromatin Remodeling Factors RT^2^ Profiler PCR Array (Cat. no. PAHS-086ZC-2) and Human Epigenetic Chromatin Modification Enzymes RT^2^ Profiler PCR Array (Cat. no. PAHS-085ZC-2) according to the manufacturer’s instructions on an ABI StepOne Plus thermocycler. Raw C_T_ values were analyzed using SABiosciences RT^2^ Profiler PCR Data Analysis software at http://pcrdataanalysis.sabiosciences.com/pcr/arrayanalysis.php and the dataset containing these results is in Additional file 1. Relative quantitation for each gene was determined by normalizing to the geometric means of five housekeeping genes (ACTB, B2M, GAPDH, HPRT1, and RPLP0) comparing SW13+ to SW13- cells using the 2^-ΔΔCt^ method.

### Western blotting

Cytosolic and nuclear protein fractions were isolated using a commercially available kit (Cell Biolabs, San Diego, CA), and histones were extracted from the remaining nuclear pellet by 1 min sonication in a sonicating water bath. Histones were separated with pre-cast tris-tricine gels (Bio-Rad) then transferred to a PVDF membrane for blotting. All primary acetyl histone antibodies were used at a 1:500 in 4 % BSA-TBS (0.01 % Tween-20). For analysis of SWI/SNF proteins 30 μg nuclear protein were loaded into pre-cast 4–20 % tris-glycine gels before being transferred to a PVDF membrane for blotting. All SWI/SNF antibodies were from a SWI/SNF complex antibody sampler kit (Cell Signal Technology) and were used at 1:1000 in 5 % NFDM-TBS (0.01 % Tween-20). All experiments and blots were performed at least in triplicate and relative expression was quantified using an Odyssey Infrared Imaging system and Image Studio v. 2.0 software (LI-COR, Lincoln, NE) using total Histone H3 protein as the invariant control.

### Statistical analysis

Differences in cell growth, percent invasion, and gene expression between SW13- and SW13+ cells were determined using a Student’s t-test. For statistical analysis of subtype switching, intensity ratio data were calibrated using a direct count of percentage of number of cells expressing vimentin over the total number of cells. As the fluorescence intensity ratio data failed the Shapiro-Wilks test for normality, it was analyzed using the Independent-Sample Kruskal-Wallis test to compare means. Western blot band densitometry was normalized to percent of total protein loaded as determined by total Histone H3 band density and analyzed by one-way ANOVA using SPSS software, version 19 (Chicago, IL). All tests were conducted at the 95 % confidence interval and presented as means ± SEM. The fraction of cells proliferating was compared between the four cell lines by means of z-test according to Forthofer et al. [18]. A post-hoc Bonferroni correction was applied to all tests to account for multiple comparisons. The z-test was carried out using Microsoft Excel 2002 (Microsoft Corp.). Statistical tests were two-sided and statistical significance was assumed if *p* <0.05.

## Results

### Characterization of SW13 subtypes

SW13+ and SW13- subtypes were isolated by dilution cloning from a sample of grade IV, primary small cell carcinoma from adrenal cortex obtained from ATCC. The subtypes are stable for at least 20 doublings, and all experiments were conducted on clones with less than 20 passages [12]. As seen in Fig. 1a, although isolated from the same tumor sample, SW13- and SW13+ subtypes have distinct cellular morphology and actin cytoskeletal structures in addition to the absence or presence of a vimentin intermediate filament network. It is noteworthy that the SW13- cells have a cortical actin cytoskeleton which is characteristic of epithelial cells while the SW13+ cells appear to be more mesenchymal-like in that they express vimentin and organize their actin into fibers. In SW13- cells, mRNA for both *VIM* and *BRM* were near the limit of threshold for detection. In contrast, mRNA expression for both *VIM* and *BRM* were approximately eight fold higher in SW13+ cells compared to SW13- cells (Fig. 1b). These results are in agreement with others [12, 13, 19].

**Fig. 2:**
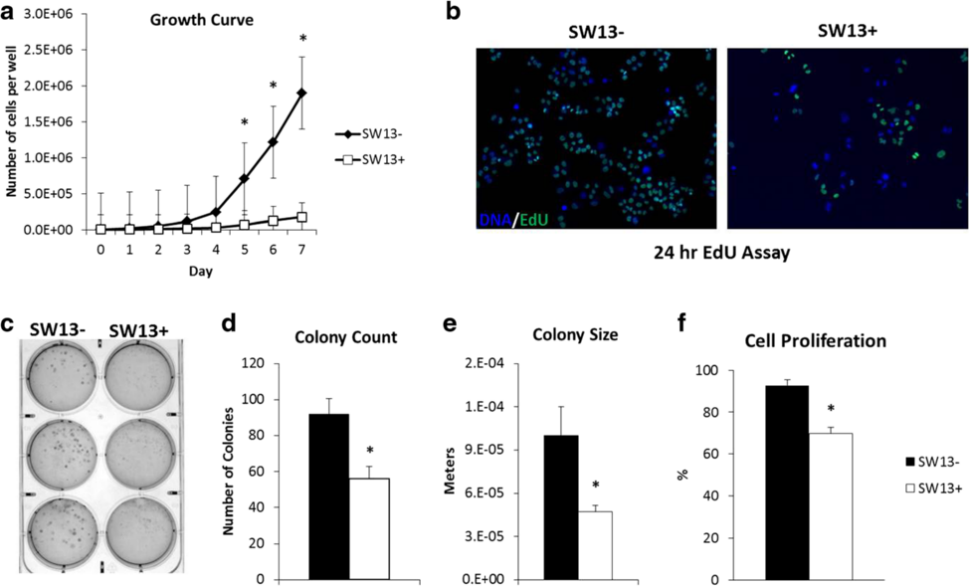
SW13- cells are more proliferative and have higher rates of anchorage independent growth than SW13+. SW13- and SW13+ cells were seeded in 6-well plates at 1 × 10^4^ cells per well and counted using a hemocytometer and trypan exclusion every 24 h for 7 days. **a** The growth rate of SW13- cells is significantly higher than SW13+ cells. **b** SW13- cells have higher rates of proliferation as determined by the higher number of EdU positive cells (*green*) per total number of cells indicated by Hoechst 33342 staining (*blue*). Images were taken using a 10× objective lens and quantitated (**f**) using ImageJ software. **c** Soft agar colony formation assays revealed SW13- cells exhibit increased rates of anchorage independent growth as indicated by (**d**) increased colony number and (**e**) increased colony size. Data are presented as mean ± SEM. *Denotes statistical difference between subtypes, *p* <0.05

With these differences characterized, we next investigated phenotypic differences between the two subtypes as they relate to oncogenic potential. Proliferation rates of each subtype were assessed by a standard growth assay in which cells were plated at equal densities and the number of cells was counted daily. The SW13- cells began their exponential growth phase between days 4 and 5 at which the difference in cell numbers between SW13- and SW13+ cells became significantly different (Fig. 2a). This trend continued through the end of the experiment at day 7, wherein the SW13- cells were continuing to increase in number rapidly while SW13+ cell numbers only continued to increase slowly. These data were confirmed by measuring cell proliferation (Fig. 2b, f) in which SW13+ had significantly reduced rates of proliferation. In addition to their higher growth rate, SW13- cells also showed a significantly greater ability to form colonies in soft agar, forming both more numerous and larger colonies (Fig. 2b, c, and d).

**Fig. 3:**
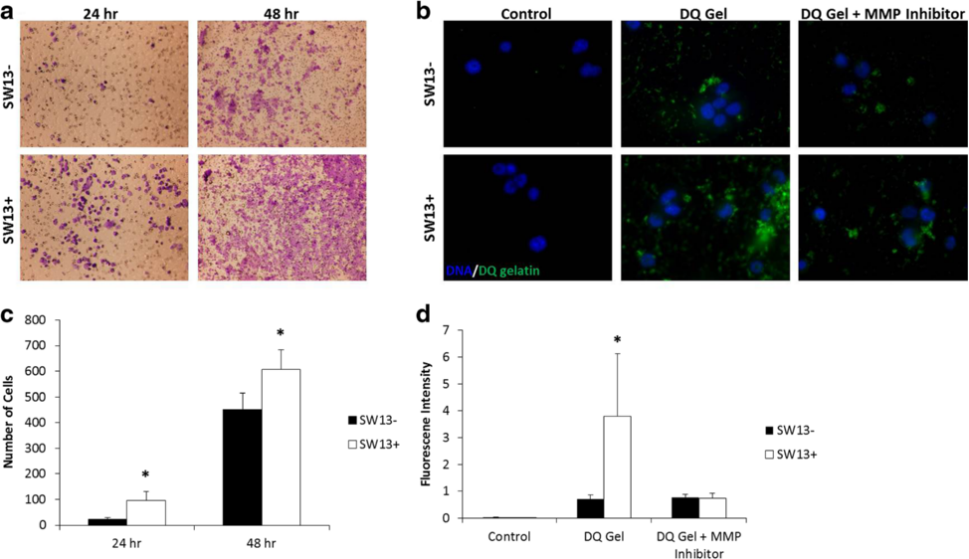
SW13+ cells display properties associated with increased rates of metastasis to a greater extent than do SW13- cells. **a** and **c** Transwell migration assays indicate SW13+ cells have increased rates of chemotaxis compared to the SW13- cells at 24 and 48 h. Images were taken using a 4× objective lens (**b** and **d**) SW13+ cells exhibited higher levels of MMP2/9 (collagenase) activity as measured by increased fluorescence after incubation with a fluorescein-quenched gelatin substrate (DQ gelatin). Addition of a broad-spectrum MMP inhibitor reduced the fluorescence signal in both subtypes. Images were taken using a 40× objective lens. Data are presented as mean ± SEM. *Denotes statistical difference between subtypes, *p* <0.05

The potential for metastasis in each subtype was then assessed by migration and MMP activity assays. Transwell migration assays indicate that the mesenchymal-like SW13+ cells which express vimentin had significantly higher numbers of cells migrating through the pores than the SW13- cells at both 24 and 48 h (Fig. 3a and c). These data are supported by results from scratch assays, where SW13+ cells appear to close a scratch faster than SW13- cells (in our hands and [20, 21]). SW13+ cells exhibited higher levels of MMP2/9 (collagenase) activity as measured by increased fluorescence after incubation with a fluorescein-quenched gelatin substrate (Fig. 3b and d). Addition of a broad-spectrum MMP peptide inhibitor reduced the fluorescence signal in both subtypes, suggesting that at least part of the increased fluorescence is specifically due to MMP activity.

### Characterization of HDAC inhibitors and subtype switching

**Fig. 4:**
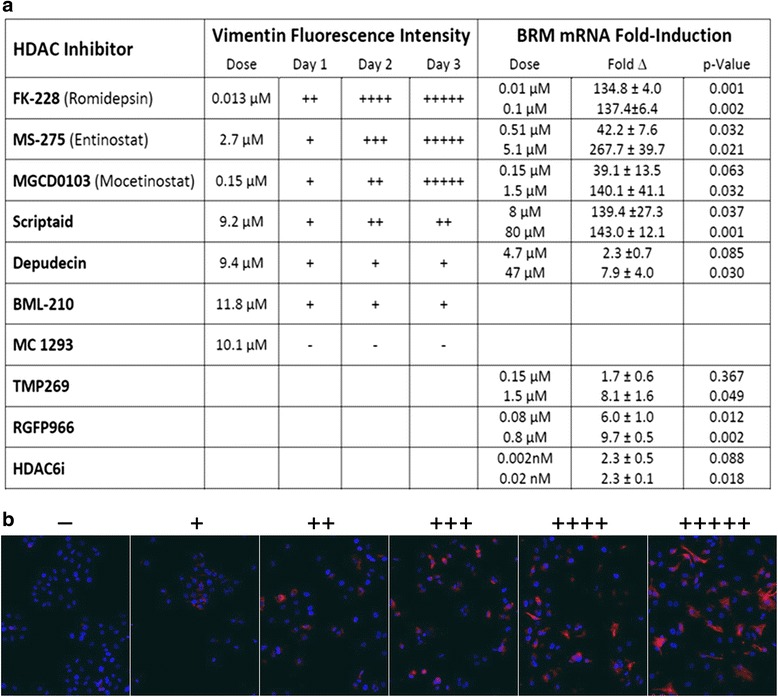
The ability of selected HDAC inhibitors to induce the switching from the SW13- to the SW13+ subtype. **a** Induction of vimentin expression was assayed at 24, 48, and 72 h using fluorescence intensity of vimentin normalized to DAPI staining at each time point (*left columns*). Dose response was measured by fold induction in *BRM* mRNA using qPCR for two doses of each inhibitor (*right columns*). **b** Representative immunofluorescence images which illustrate the range of vimentin induction in panel (**a**). Scale ranges from no detectable vimentin immunofluorescence signal (−) to + representing <20 %, ++ representing 20–40 %, +++ representing 41–60 %, ++++ representing 61–80 %, and +++++ representing 81–100 % of maximum vimentin normalized to DAPI staining. Images were taken using a 10× objective lens. Data (right hand column of panel **a**) are presented as mean ± SEM

Although it is unclear how the SW13- subtype “spontaneously” transforms into the SW13+ subtype, it is well established that HDAC inhibition can induce an SW13- to SW13+ subtype switch [12, 13, 19]. As the efficacy of various HDAC inhibitors has not previously been investigated, we initially screened HDAC inhibitors to focus subsequent analyses. We tested doses based on the reported IC_50_ for each HDAC inhibitor. However, as these values are typically based on in vitro assays on recombinant proteins and difficult to translate to cell-based assays, a broad range of concentrations were tested. Immunofluorescence analysis of vimentin intensity, normalized for cell number as measured by intensity of DAPI staining, was determined using SW13- cells after incubation with an HDACi for 1, 2, or 3 days. These data are summarized in Fig. 4a while the immunofluorescence photographs that illustrate how these data were compiled are shown in Fig. 4b. The broad-spectrum inhibitor Scriptaid was less efficient at inducing vimentin expression than MS-275 (Entinostat), FK228 (Romidepsin), or MGCD0103 (Mocetinostat). These HDAC inhibitors are reported to be potent HDAC1 inhibitors with some activity against other HDACs [22–24] and on the basis of these data, we focused our attention on HDAC1 as the most likely enzyme to be involved in the SW13- to SW13+ subtype switch.

An alternate HDACi screen, focusing on the induction of *BRM* mRNA after 24 h of treatment, reveals similar trends (Fig. 4a, right hand columns). MGCD0103 and MS-275 showed a clear dose-response effect at the two concentrations tested. However, even at the lowest dose tested, FK228 yielded *BRM* expression levels equivalent to the highest dose of the other two inhibitors. In this assay, Scriptaid yielded similar levels of *BRM* induction as the more specific HDAC1 inhibitors.

Treatment with HDACi which are not specific for HDAC1 were less effective in inducing the expression of vimentin and BRM. BML-210 (reported to have the highest affinity for HDAC3 [25, 26]), and MC1293 (reported to bind HDAC6 and HDAC10 and to HDAC1 with lower affinity [26]) are minimally effective or ineffective in inducing vimentin expression. Depudecin treatment led to minimal expression of vimentin and BRM but this HDAC inhibitor has only been tested against recombinant HDAC1 [27] so that its specificity has not been determined. TMP269 (reported to be a Class IIA HDAC inhibitor specific for HDACs 4, 5, 7, and 9 [28]), RGFP966 (an HDACi reported to be specific for HDAC3 [29]), and HDAC6i (characterized as an HDAC6 specific inhibitor [30]) were also tested. Although some of these yielded statistically significant increases in BRM at one or both tested doses, the fold increases were 1–2 orders of magnitude lower than the HDAC inhibitors which have been reported to be more specific towards HDAC1.

**Fig. 5:**
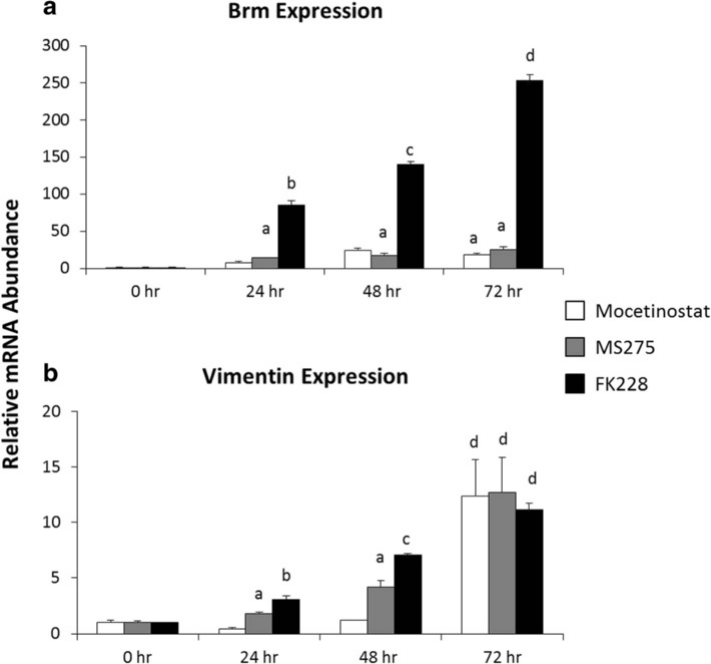
Inhibitors of HDAC1 increase BRM and VIM expression in a time-dependent manner. Total RNA was isolated from untreated SW13- cells (0 h), and SW13- cells treated with 0.15 μM MGCD0103, 0.51 μM MS-275, or 2 nM FK228 for 24, 48, and 72 h. Fold change in (**a**) *BRM* and (**b**) *VIM* mRNA expression from 0 h was determined by real-time PCR analysis using the 2^-ΔΔCt^ method normalized to GAPDH. Data are presented as mean ± SEM. Superscripts indicate statistical significance, *p* <0.05. Treatments that share the same superscripts are not significantly different from one another

We next followed the change in *BRM* and *VIM* expression over time with the three most effective HDAC inhibitors. These data integrate the vimentin protein data over time (Fig. 4a, b) with the *BRM* expression data at a single time point with multiple doses of HDACi. MGCD0103, MS-275 and FK228 yielded similar results for *VIM* expression (Fig. 5b). However, FK228 treatment at 1nM yielded significantly higher increases in *BRM* expression (Fig. 5a). Taken together, these results strongly suggest that inhibition of HDAC1 results in the most effective re-expression of *BRM* and *VIM*.

### Differing efficacy of FK228 and MS-275 promoting subtype switching

**Fig. 6:**
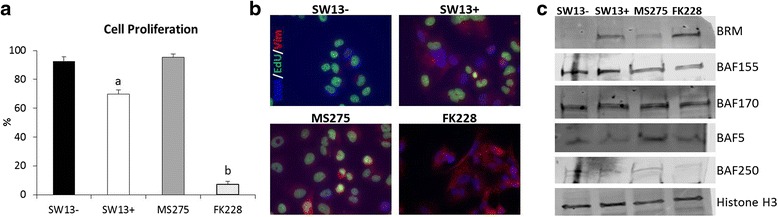
HDAC1 inhibitors reduce cell proliferation and promote restoration of VIM and BRM protein to varying degrees. **a** SW13- and SW13+ cells as well as SW13- cells treated with either 0.51 μM MS-275 or 2 nM FK228 for 24 h were labeled with EdU for 24 h to determine proliferation rates. **b** Representative images of two independent experiments performed in triplicate are shown. **c** Nuclear protein was isolated from SW13- and SW13+ cells, as well as from SW13- cells which had been treated with either 0.51 μM MS-275 or 2 nM FK228 for 24 h and the expression of SWI/SNF protein components were examined by western blot. Total histone H3 was used as a loading control. Data are presented as mean ± SEM. Superscripts indicate statistical significance, *p* <0.05

As shown in Fig. 5, FK228 is more effective at inducing BRM protein expression than either of the other HDAC1-specific inhibitors. To investigate this difference further, we compared the efficacy of FK228 and MS-275 after 24 h of treatment with respect to proliferation, vimentin expression, and induction of BRM protein (Fig. 6). At this time point, FK228 reduced proliferation to ~8 % of that seen in untreated SW13+ with significant vimentin and BRM expression. In contrast, treatment with MS-275 did not significantly alter proliferation and resulted in less vimentin and BRM expression. Neither HDACi treatment appears to significantly alter expression of the four other subunits of the SWI/SNF complex which were examined.

### HDACi induced changes & comparison to SW13 subtypes

Although transient HDAC inhibition initiates the switch from SW13- to SW13+ subtypes, the resulting epigenetic switch between subtypes is stable for greater than 20 doublings [12]. Therefore, we hypothesized that HDAC inhibition initiated changes in SW13- chromatin structure and gene expression which then lead to the propagation of a stable SW13+ subtype after the removal of the HDACi. To probe these changes, we next addressed histone modification and differential gene expression during HDAC treatment and in the stable subtypes.

**Fig. 7:**
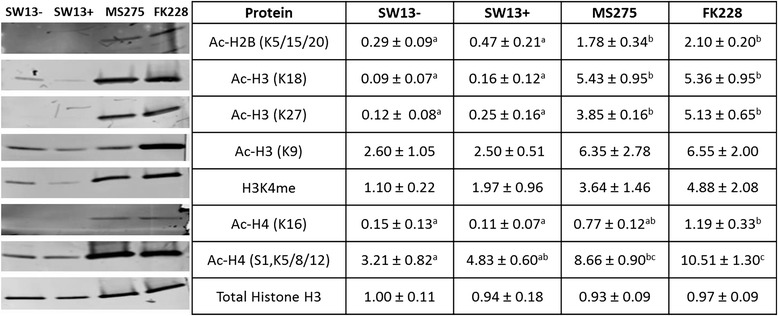
Histone acetylation in stable SW13- and SW13+ subtypes and following treatment with the HDAC inhibitors MS-275 and FK228. Pure histones were isolated from SW13- and SW13+ cells, as well as from SW13- cells which had been treated with either 0.51 μM MS-275 or 2 nM FK228 for 24 h. Histones were separated on tris-tricine gels and transferred to a PVDF membrane for blotting and assessment of histone modifications. Total histone H3 was used as a loading control. Data are presented as mean ± SEM. Superscripts indicate statistical significance, *p* <0.05. Within a given row cell types that share the same superscripts are not significantly different from one another

We assayed the effects of HDACi treatment on major modified histone lysine residues on H2B, H3, and H4. As we hypothesize that the SW13- to SW13+ subtype switch involves induction of gene expression, we also included tri-methylation of H3K4 as this is strongly associated with gene activation. Although HDACs have many non-histone targets, we focused on histone modifications associated with gene activation as the subtype switch involves changes in gene expression. As shown in Fig. 7, western blot analysis of purified histones revealed that treatment with FK228 and MS-275 both result in very elevated levels of all histone modifications tested. Although HDACi treatment initiates the switch, SW13+ cells do not have globally higher levels of acetylation than SW13-.

### Characterization of epigenetic regulators in SW13 subtypes

**Fig. 8:**
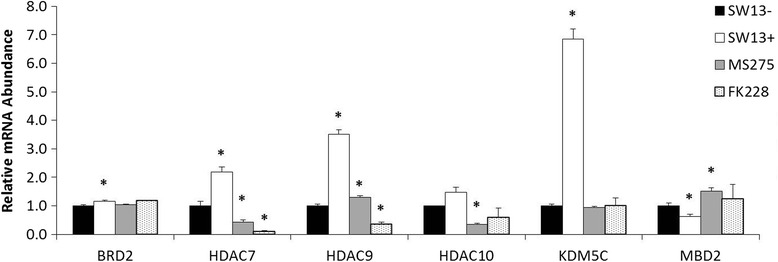
Validation of select genes related to epigenetic chromatin modification and remodeling by qPCR. Relative expression of a total of bromodomain containing 2 (BRD2), HDAC7, HDAC9, HDAC10, lysine (K)-specific demethylase 5c (KDM5C), and methyl-CpG binding domain protein 2 (MBD2) was examined in stable SW13- and SW13+ subtypes and SW13- cells which had been treated for 24 h with either 0.51 μM MS-275 or 2 nM FK228. Fold-regulation was determined by the 2^-ΔΔCT^ method using GAPDH as the invariant control. Data are presented as mean ± SEM. *Denotes statistical difference from SW13-, *p* <0.05

It is clear that HDACi treatment yields changes in gene expression during the first 24 h of treatment (e.g. BRM and VIM in Figs. 4 and 5) which are characteristic of SW13+ cells isolated by limiting dilution cloning. We hypothesized that other genes must be differentially expressed during this period which alters chromatin structure in order to generate the observed stable subtypes after removal of HDACi. In an effort to uncover factors that may contribute to the maintenance of phenotypic differences between the two subtypes, we screened mRNA isolated from the stable SW13- and SW13+ subtypes for differential expression of genes involved in chromatin modification and remodeling using pathway-focused qRT-PCR arrays (see Additional file 1). Differential expression of six of these genes was validated by qPCR (Fig. 8).

The differentially expressed genes appear to be involved in multiple levels of epigenetic regulation. We saw an increased expression of genes involved in histone acetylation, like *HDAC7*, *9, 10*, or in the binding of acetyl-histones, such as *BRD2*. Additionally, the methyl CpG DNA binding protein *MDB2* is downregulated approximately two-fold although we did not detect any differences in global levels of DNA methylation (data not shown). *KDM5C*, specific for the demethylation of H3K4me2 and H3K4me3, showed increased expression. We next asked if these genes were differentially expressed in the same manner during HDACi treatment. As shown in Fig. 8, the expression pattern during treatment showed differences during treatment when compared to either subtype. These data suggest subtype determination is controlled at multiple levels and that epigenetic factors not involved in the subtype switch may be involved in the maintenance of stable subtype phenotype.

## Discussion

Epigenetically plastic cancer cells represent a major obstacle for effective treatment regimes. The ability of distinct SW13 subtypes to transition between two subtypes with differing oncogenic properties represents an opportunity to explore mechanisms of epigenetic regulation. The transition from the SW13- subtype with higher rates of proliferation and colony formation to the more metastatic SW13+ is initiated by HDAC inhibition but also involves differential expression of chromatin remodeling enzymes and factors which read these chromatin marks, implicating multiple levels of epigenetic regulation.

Distinct SW13 subtypes which differ in BRM and vimentin expression have been previously described [1, 12] but have been further characterized in this work. We found that *BRM* deficient SW13- cells have a significantly higher growth rate than SW13+ cells, which is in agreement with the findings of others who have reported that ex vivo cultures of embryonic fibroblasts from BRM knockout mice also grow significantly faster than their wild-type controls [31]. Similar to the growth rate findings, the colony forming capacity of *BRM* deficient SW13- cells was also significantly higher than that of *BRM* expressing SW13+ cells, but based on the vast differences in growth rates we could not determine if this was simply due to the differences in overall cell numbers. However, similar observations have been noted in SW13- cells that were virally transfected to overexpress BRM, which also had significantly reduced anchorage independent growth [19].

While inducing expression of BRM to slow cell growth might be considered desirable, increased expression of vimentin is associated with a more motile and invasive mesenchymal-like cancer cell phenotype [32]. Here, we demonstrate that the vimentin expressing SW13+ cells had significantly higher invasive capacity than the SW13- cells. We also show that this is likely due to increased MMP expression and activity, as SW13+ cells displayed higher amounts of collagenase breakdown, but treatment with an MMP inhibitor decreased this activity. As SW13+ cells also express two markers associated with cancer stem cells (CD44 and cMET [12]), they may have additional attributes which contribute to oncogenesis.

The inhibitor data presented here suggests a role for inhibition of HDAC1 in promoting the conversion of SW13- to SW13+ cells. However, given the complex role of HDACs within the cell, it is unclear how HDAC1 may trigger the subtype switch. First, HDACs have many non-histone targets [33–35] and HDAC inhibitors are likely to increase lysine acetylation on many other cellular proteins. Second, HDACs form multiple complexes with other proteins including other HDACs so it is unclear how HDAC inhibitors might affect the activity of other proteins in the complex (reviewed in [14]). Third, specificities of HDACs are typically tested using recombinant human HDAC protein and a general HDAC peptide substrate. It is unclear how these data translate to in vivo activity inhibition. Fourth, although HDAC1 itself does not appear to be differentially expressed, the expression level of several other HDACs change during the first 24 h of treatment, suggesting that alterations in acetylation as a result of HDACi treatment is complex. Despite these factors which complicate interpretation, it is clear that addition of inhibitors which target HDAC1 induce the switch between SW13- and SW13+ subtypes.

Although not gene specific, the dramatic increase in histone modifications observed after the addition of HDAC inhibitors include those known to be deacetylated by HDAC1. This includes several modifications associated with gene activation (including H3K9, H4K16 [36]) but not acetylation of H3K56, which is not associated with gene activation ([37–39] reviewed in [40, 41]). In addition, others have seen that inhibition of histone deacetylases alter histone methylation levels [42].

Although modification of histone acetylation and methylation induce subtype switching, other factors may be responsible for maintaining the subtypes. It is possible that differentially expressed chromatin remodeling factors and epigenetic chromatin modification enzymes, related to several of the histone modifications, may be responsible for maintaining the SW13+ subtype for >20 generations. As suggested by Fig. 8, these maintenance factors may be different from those involved in the HDACi-mediated switch. As SW13+ cells isolated by dilution cloning and SW13- cells after the removal of HDACi appear indistinguishable, the significance of the differential gene expression during HDACi treatment is unclear.

Thus, the switch between the SW13 subtypes is likely to involve both chromatin remodeling and histone modification, two important mechanisms by which chromatin structure and gene activation are regulated. After restoration of BRM expression, SW13+ cells have a functional SWI/SNF complex [13]. The SWI/SNF complex has been shown to both reposition and slide nucleosomes [43, 44] and interact with modified histones [45–47], activities predominately associated with gene activation. However, the SWI/SNF complex, in association with other complexes has also been associated with gene repression [48–50]. In the case of glucocorticoid receptor signaling to recruit the SWI/SNF chromatin remodeling complex, active BRM [13] or BRG1 [51–53] is required and SWI/SNF recruitment results in both gene activation and silencing [51]. This has led to models in which transcription factors recruit chromatin remodeling complexes [51, 53, 54]. In addition, the SWI/SNF complex has been linked to changes in DNA methylation affecting the expression of CD44 [55], suggesting that others have seen similar relationships between SWI/SNF activity, DNA modification, and changes in phenotypically important gene expression.

Elucidation of the subtype switch mechanism may have profound implications for the treatment of multiple types of cancer. Although HDAC inhibitors have been approved for the treatment of lymphomas, they have not proved beneficial for the treatment of solid tumors. As shown here, HDAC inhibitors induce a metastatic phenotype. To date, there is no known trigger for the SW13+ to SW13- subtype. Further characterization of the epigenetic differences between the two subtypes and the mechanisms governing this subtype switch are of intense interest.

## Conclusions

HDAC1 inhibition most effectively induces a switch from the more proliferative SW13- to the more metastatic SW13+ subtype which expresses *BRM* and *VIM*. The switch occurs in stages with *BRM* mRNA elevating earlier than vimentin. The data are consistent with a mechanism in which HDAC inhibitors initiate subtype conversion by inducing hypermodified histone residues and changes in gene expression mediated by a now functional SWI/SNF complex, followed by subtype switch stabilization via changes in chromatin remodeling factors and chromatin epigenetic enzymes.

## Declarations

### Ethics approval and consent to participate

Not applicable.

### Consent for publication

Not applicable.

### Availability of data and materials

The dataset supporting the conclusions of this article is included within the article (and its additional file).

## Additional file

Additional file 1: Human Epigenetic Chromatin Modification Enzymes and Epigenetic Chromatin Remodeling Factors RT^2^ Profiler™ PCR array analysis of gene expression patterns of SW13+ cells compared to SW13- cells. The expression patterns of 84 different genes related to (a) chromatin modification enzymes and 84 genes related to (b) chromatin remodeling factors are shown. Fold-change values >1 indicate an up-regulation; while fold-change values <1 indicate a down-regulation. Fold-change values >2 are considered significant. Comments with “A” indicate that the average cycle threshold (C_T_) value is relatively high (>30) in either the control or the test sample and is reasonably low in the other sample (<30), suggesting that the actual fold-change value is at least as large as the calculated and reported fold-change results. Comments with “B” indicate that the average C_T_ value is relatively high (>30), indicating that its relative expression level is low in both control and test samples, and the p value for the fold-change is either unavailable or relatively high (*p* >0.05). Comments with “C” indicate that the average C_T_ value is either not determined or greater than the defined cut-off value (C_T_ ≥35) in both samples, indicating that its expression was undetected, thus rendering this fold-change result erroneous and uninterpretable. (DOCX 18 kb)

## Abbreviations

BRD2: bromodomain containing 2
BRG1 (SMARCA4): Brahma related gene 1
BRM (SMARCA2): Brahma
HDACi: histone deacetylase inhibitor
KDM5C: lysine (K)-specific demethylase 5C
MBD2: methyl-CpG binding domain protein 2
SWI/SNF: SWItch/Sucrose NonFermentable
TSA: trichostatin A
