# Supplementary material for: Epigenetically maintained SW13+ and SW13- subtypes have different oncogenic potential and convert with HDAC1 inhibition
Source: BMC Cancer. 2016 May 17;16:316. doi: 10.1186/s12885-016-2353-7 (PMC4870788; doi:10.1186/s12885-016-2353-7)
Supplement: Additional file 1: — Human Epigenetic Chromatin Modification Enzymes and Epigenetic Chromatin Remodeling Factors RT2 Profiler™ PCR array analysis of gene expression patterns of SW13+ cells compared to SW13- cells. The expression patterns of 84 different genes related to (a) chromatin modification enzymes and 84 genes related to (b) chromatin remodeling factors are shown. Fold-change values >1 indicate an up-regulation; while fold-change values <1 indicate a down-regulation. Fold-change values >2 are considered significant. Comments with “A” indicate that the average cycle threshold (CT) value is relatively high (>30) in either the control or the test sample and is reasonably low in the other sample (<30), suggesting that the actual fold-change value is at least as large as the calculated and reported fold-change results. Comments with “B” indicate that the average CT value is relatively high (>30), indicating that its relative expression level is low in both control and test samples, and the p value for the fold-change is either unavailable or relatively high (p >0.05). Comments with “C” indicate that the average CT value is either not determined or greater than the defined cut-off value (CT ≥35) in both samples, indicating that its expression was undetected, thus rendering this fold-change result erroneous and uninterpretable. (DOCX 18 kb) [file 12885_2016_2353_MOESM1_ESM.docx]

**Additional file 1**: Human Epigenetic Chromatin Modification Enzymes and Epigenetic Chromatin Remodeling Factors RT² Profiler™ PCR array analysis of gene expression patterns of SW13+ cells compared to SW13- cells. The expression patterns of 84 different genes related to (a) chromatin modification enzymes and 84 genes related to (b) chromatin remodeling factors are shown. Fold-change values > 1 indicate an up-regulation; while fold-change values < 1 indicate a down-regulation. Fold-change values > 2 are considered significant. Comments with “A” indicate that the average cycle threshold (C_T_) value is relatively high (> 30) in either the control or the test sample and is reasonably low in the other sample (< 30), suggesting that the actual fold-change value is at least as large as the calculated and reported fold-change results. Comments with “B” indicate that the average C_T_ value is relatively high (> 30), indicating that its relative expression level is low in both control and test samples, and the p value for the fold-change is either unavailable or relatively high (p > 0.05). Comments with “C” indicate that the average C_T_ value is either not determined or greater than the defined cut-off value (C_T_ ≥ 35) in both samples, indicating that its expression was undetected, thus rendering this fold-change result erroneous and uninterpretable.

**Additional File 1a. Human Epigenetic Chromatin Modification Enzymes RT² Profiler™ PCR array analysis of gene expression patterns SW13+ cells compared to SW13- cells**

|  | Layout | 1 | 2 | 3 | 4 | 5 | 6 | 7 | 8 | 9 | 10 | 11 | 12 |
| --- | --- | --- | --- | --- | --- | --- | --- | --- | --- | --- | --- | --- | --- |
| A | Genes | ASH1L | ATF2 | AURKA | AURKB | AURKC | CARM1 | CYDL | CIITA | CSRP2BP | DNMT1 | DNMT3A | DNMT3B |
|  | Fold-change | -1.1542 | -1.1307 | -1.2094 | -.5529 | -1.0778 | -1.0346 | -1.1261 | -1.5552 | -1.3618 | 1.0476 | 1.8751 | 1.2666 |
|  | Comments | OKAY | OKAY | OKAY | OKAY | B | OKAY | OKAY | B | OKAY | OKAY | OKAY | OKAY |
| B | Genes | DOT1L | DZIP3 | EHMT2 | ESCO1 | ESCO2 | HAT1 | HDAC1 | HDAC10 | HDAC11 | HDAC2 | HDAC3 | HDAC4 |
|  | Fold-change | 1.0524 | -1.2311 | -1.1602 | -1.1828 | -1.5799 | -1.9218 | -1.086 | 2.117 | 1.8239 | 1.1155 | -1.0187 | 1.0579 |
|  | Comments | OKAY | OKAY | OKAY | OKAY | OKAY | OKAY | OKAY | OKAY | OKAY | OKAY | OKAY | OKAY |
| C | Genes | HDAC5 | HDAC6 | HDAC7 | HDAC8 | HDAC9 | KAT2A | KAT2B | KAT5 | KAT6A | KAT6B | KAT7 | KAT8 |
|  | Fold-change | -1.1536 | 1.2954 | 2.1541 | -1.0834 | 3.3807 | 1.2845 | 1.7847 | 1.5006 | 1.0991 | 1.8266 | 1.9638 | 1.1224 |
|  | Comments | OKAY | OKAY | OKAY | OKAY | OKAY | OKAY | OKAY | OKAY | OKAY | OKAY | OKAY | OKAY |
| D | Genes | KDM1A | KDM4A | KDM4C | KDM5B | KDM5C | KDM6B | MBD2 | KMT2A | KMT2C | KMT2E | MYSM1 | NCOA1 |
|  | Fold-change | -2.2798 | -1.2406 | 1.008 | 1.1985 | 4479.035 | 1.6732 | -1.8484 | 1.0184 | -1.3758 | -2.5206 | 1.608 | 1.8152 |
|  | Comments | OKAY | OKAY | OKAY | OKAY | A | OKAY | OKAY | OKAY | OKAY | OKAY | OKAY | OKAY |
| E | Genes | NCOA3 | NCOA6 | NEK6 | NSD1 | PAK1 | PRMT1 | PRMT2 | PRMT3 | PRMT5 | PRMT6 | PRMT7 | PRMT8 |
|  | Fold-change | -1.2582 | -1.7347 | 1.8027 | 1.3316 | 1.4174 | -1.1736 | 1.8008 | 1.2767 | -1.0411 | -1.2482 | 1.2712 | -1.0671 |
|  | Comments | OKAY | OKAY | OKAY | OKAY | OKAY | OKAY | OKAY | OKAY | OKAY | OKAY | OKAY | C |
| F | Genes | RNF2 | RNF20 | RPSKA3 | RPSKA5 | SETD1A | SETD1B | SETD2 | SETD3 | SETD4 | SETD5 | SETD6 | SETD7 |
|  | Fold-change | -1.4427 | 1.1993 | -1.2557 | 1.2566 | 1.2851 | 1.7287 | 1.6116 | 1.359 | 1.4715 | -1.5732 | 2.4716 | 2.8736 |
|  | Comments | OKAY | OKAY | OKAY | OKAY | OKAY | OKAY | OKAY | OKAY | OKAY | OKAY | B | OKAY |
| G | Genes | SETD8 | SETDB1 | SETDB2 | SMYD3 | SUV39H1 | SUV420H1 | UBE2A | UBE2B | USP16 | USP21 | USP22 | WHSC1 |
|  | Fold-change | -1.1805 | 1.0303 | -2.1071 | 1.4426 | 1.6824 | 1.4345 | 1.4614 | 1.1805 | 1.4684 | 1.4182 | 2.078 | 1.4119 |
|  | Comments | OKAY | OKAY | OKAY | OKAY | OKAY | OKAY | OKAY | OKAY | OKAY | OKAY | OKAY | OKAY |

**Additional File 1b. Human Epigenetic Chromatin Remodeling Factors RT² Profiler™ PCR array analysis of gene expression patterns SW13+ cells compared to SW13- cells**

|  | Layout | 1 | 2 | 3 | 4 | 5 | 6 | 7 | 8 | 9 | 10 | 11 | 12 |
| --- | --- | --- | --- | --- | --- | --- | --- | --- | --- | --- | --- | --- | --- |
| A | Genes | ARID1A | ASXL1 | BAZ1A | BAZ1B | BAZ2A | BAZ2B | BMI | BPTF | BRD1 | BRD2 | BRD3 | BRD4 |
|  | Fold-change | 1.055 | 1.099 | -1.0998 | 1.0021 | 1.5238 | 1.2101 | 1.3643 | 1.6658 | 2.2504 | 2.0408 | 1.9095 | 2.9976 |
|  | Comments | OKAY | OKAY | OKAY | OKAY | OKAY | OKAY | OKAY | OKAY | OKAY | OKAY | OKAY | OKAY |
| B | Genes | BRD7 | BRD8 | BRDT | BRPF1 | BRPF3 | BRWD1 | BRWD3 | CBX1 | CBX3 | CBX4 | CBX5 | CBX6 |
|  | Fold-change | 1.1027 | 1.1909 | -1.2116 | 1.265 | 1.6569 | 1.2511 | 1.1936 | 1.2393 | 1.191 | 1.5129 | 1.5929 | 3.5167 |
|  | Comments | OKAY | OKAY | C | OKAY | OKAY | OKAY | OKAY | OKAY | OKAY | OKAY | OKAY | OKAY |
| C | Genes | CBX7 | CBX8 | CDYL | CDYL2 | CHD1 | CHD2 | CHD3 | CHD4 | CHD5 | CHD6 | CHD7 | CHD8 |
|  | Fold-change | 1.293 | -1.0339 | 1.2038 | 13.5078 | 1.1956 | 1.44 | -1.2171 | 1.0062 | 1.1001 | 1.4677 | -1.4633 | 1.4666 |
|  | Comments | OKAY | OKAY | OKAY | A | OKAY | OKAY | OKAY | OKAY | OKAY | OKAY | OKAY | OKAY |
| D | Genes | CHD9 | CTBP1 | CTBP2 | CTCF | EED | EZH2 | HINFP | ING1 | ING2 | ING3 | ING4 | ING5 |
|  | Fold-change | -1.1788 | 1.0242 | 1.4553 | 1.1079 | 1.0584 | -1.1146 | 1.0645 | 1.0294 | -1.027 | -1.0045 | 1.0395 | -1.2516 |
|  | Comments | OKAY | OKAY | OKAY | OKAY | OKAY | OKAY | OKAY | OKAY | OKAY | OKAY | OKAY | OKAY |
| E | Genes | INO80 | MBD1 | MBD2 | MBD3 | MBD4 | MECP2 | MTA1 | MTA2 | NAB2 | NSD1 | PBRM1 | PCGF1 |
|  | Fold-change | -1.1858 | -1.4375 | -2.0395 | 1.2099 | 1.1166 | 1.2376 | -1.379 | -1.0344 | 1.097 | -1.1267 | -1.3388 | -1.3509 |
|  | Comments | OKAY | OKAY | OKAY | OKAY | OKAY | OKAY | OKAY | OKAY | OKAY | OKAY | OKAY | OKAY |
| F | Genes | PCGF2 | PCGF3 | PCGF5 | PCGF6 | PHC1 | PCH2 | PHF1 | PHF13 | PHF2 | PHF21A | PHF21B | PHF3 |
|  | Fold-change | -1.123 | 1.4788 | 2.0911 | -1.1265 | 1.0362 | -1.0969 | -1.0024 | -1.0263 | -1.3423 | -1.1302 | -2.6225 | -1.39 |
|  | Comments | OKAY | B | OKAY | OKAY | OKAY | OKAY | OKAY | OKAY | OKAY | OKAY | OKAY | OKAY |
| G | Genes | PHF5A | PHF6 | PHF7 | RING1 | RNF2 | SMARCA2 | SMARCA4 | SPEN | SUZ12 | TRIM27 | WDR11 | ZMYND8 |
|  | Fold-change | -1.3664 | 2.3632 | -1.3841 | 1.1103 | -1.1539 | 3.35506 | -1.2251 | -1.2971 | -1.3466 | -1.2516 | 1.0023 | 1.203 |
|  | Comments | OKAY | OKAY | OKAY | OKAY | OKAY | A | OKAY | OKAY | OKAY | OKAY | OKAY | OKAY |
